# ‘Reliability of new poly (lactic-co-glycolic acid) membranes treated with oxygen plasma plus silicon dioxide layers for pre-prosthetic guided bone regeneration processes’

**DOI:** 10.4317/medoral.21512

**Published:** 2017-02-04

**Authors:** Gabriel Castillo-Dalí, Raquel Castillo-Oyagüe, Antonio Batista-Cruzado, Carmen López-Santos, Agustín Rodríguez-González-Elipe, Jean-Louis Saffar, Christopher D. Lynch, José-Luis Gutiérrez-Pérez, Daniel Torres-Lagares

**Affiliations:** 1 Dr. Biol, PhD. DDS. DMD, PhD. DDS, PhD. Department of Stomatology, Faculty of Dentistry, University of Seville (US), C/ Avicena, s/n, 41009, Seville, Spain; 2 DDS, PhD. Department of Buccofacial Prostheses, Faculty of Dentistry, Complutense University of Madrid (UCM), Pza. Ramón y Cajal, s/n, 28040, Madrid, Spain; 3 Dr. Phys. PhD. DDS. Institute of Materials Science of Seville (CSIC-University of Seville), C/ Américo Vespucio, 49, 41092, Seville, Spain; 4 DDS, PhD Faculté de Chirurgie Dentaire, Université Paris V – Descartes, rue Maurice Arnoux, no. 1, 92120, Montrouge, Paris, Franc; 5 BDS, PhD, MFD, FDS (Rest Dent), FACD, FHEA. School of Dentistry, Cardiff University, CF14 4XY, Cardiff, Wales, U.K

## Abstract

**Background:**

The use of cold plasmas may improve the surface roughness of poly(lactic-co-glycolic) acid (PLGA) membranes, which may stimulate the adhesion of osteogenic mediators and cells, thus accelerating the biodegradation of the barriers. Moreover, the incorporation of metallic-oxide particles to the surface of these membranes may enhance their osteoinductive capacity. Therefore, the aim of this paper was to evaluate the reliability of a new PLGA membrane after being treated with oxygen plasma (PO2) plus silicon dioxide (SiO2) layers for guided bone regeneration (GBR) processes.

**Material and Methods:**

Circumferential bone defects (diameter: 11 mm; depth: 3 mm) were created on the top of eight experimentation rabbits’ skulls and were randomly covered with: (1) PLGA membranes (control), or (2) PLGA/PO2/SiO2 barriers. The animals were euthanized two months afterwards. A micromorphologic study was then performed using ROI (region of interest) colour analysis. Percentage of new bone formation, length of mineralised bone, concentration of osteoclasts, and intensity of ostheosynthetic activity were assessed and compared with those of the original bone tissue. The Kruskal-Wallis test was applied for between-group com Asignificance level of a=0.05 was considered.

**Results:**

The PLGA/PO2/SiO2 membranes achieved the significantly highest new bone formation, length of mineralised bone, concentration of osteoclasts, and ostheosynthetic activity. The percentage of regenerated bone supplied by the new membranes was similar to that of the original bone tissue. Unlike what happened in the control group, PLGA/PO2/SiO2 membranes predominantly showed bone layers in advanced stages of formation.

**Conclusions:**

The addition of SiO2 layers to PLGA membranes pre-treated with PO2 improves their bone-regeneration potential. Although further research is necessary to corroborate these conclusions in humans, this could be a promising strategy to rebuild the bone architecture prior to rehabilitate edentulous areas.

**Key words:**Guided bone regeneration (GBR), poly(lactic-co-glycolic acid) (PLGA), membrane; oxygen plasma (PO2), nanocomposite, silicon dioxide layers.

## Introduction

Silicon is an essential trace element in humans and one of the twelve major elements that are present in the organisms. Nowadays, Tissue engineering, Biomedicine, and Odontology are working to design new biomaterials that can be modified with this element at the nanometric scale to mimic the original tissues’ structure in addition to providing a stable support for the extracellular matrix (1-3). These investigations are revolutionizing the classical techniques encompassed in the concept of guided bone regeneration (GBR) that were traditionally based on the use of physical barriers to prevent the gingival epithelium and connective tissue cells from invading the bone cavity during the healing process (4), and even the scattering of the graft particles in surgical manipulations (5).

PLGA is a synthetic biomaterial formed by poly(lactic-co-glycolic) acid copolymer that can be resorbed by the human body without generating an immune rejection reaction (6). PLGA membranes have demonstrated a correct efficiency, adhesion, and vascularisation of the surrounding tissues (1). Although these polymers are hydrophobic (3), which decreases their mechanical resistance, facilitates the release of acid residues, diminishes the pH, and favours the bacterial proliferation and inflammatory responses; the functionalisation of their surfaces with cold plasma may fairly overcome these drawbacks (2,7). On the one hand, the use of cold plasmas may improve the surface roughness of PLGA membranes, which may stimulate the adhesion of osteogenic mediators and cells, thus accelerating the biodegradation of the barriers (2,7,8). On the other hand, the incorporation of thin layers, such as metallic oxides, may optimise the osteoinductive capacity of these membranes. This may facilitate the adherence of osteblasts and the functional differentiation by regulating specific genes that participate in the neogenesis of the mineralised matrix (2,9-11).

Our research describes a new method based on the incorporation of silicon dioxide (SiO2) layers (through ‘dry way’ apposition) onto PLGA membranes modified with oxygen plasma (PO2) to increase the bone-regeneration potential of this biomaterial. Even though the pilot study included a group of PLGA/PO2/SiO2 barriers (3), the present investigation represents a major advance, as the pilot evaluation only showed data trends without statistical significance. In the current study, bigger bone defects were created (with double diameter). This increased the difficulty for filling the defects and made the histological differences between groups more evident. A greater sample size of rabbits was used (almost three times larger than that of the pilot trial). Unlike in the pilot experiment, a double marking technique was applied (with calcein) to assess the length of mineralised bone formed in the grown defects, and two essential parameters of bone regeneration were compared with those of the original bone tissue (i.e., percentage of new bone formation, and concentration of osteoclasts/mm2). Many authors have also utilised parietal bone of rabbits for related purposes (3,12-14). The calvarial bone and the maxillary/mandible bone have comparable membranous embryological origins, as both are derived from the neural crest (13). Nonetheless, the findings of animal studies that use parietal bone should be always considered as a previous necessary step to be corroborated in prospective investigations that use human maxillary bone and long healing periods.

The aim and novelty of this prospective cohort animal study were, therefore, to in vivo evaluate the bone-regeneration capacity of PLGA membranes treated with PO2 and sputtered with nanometric layers of SiO2, which may act as osteogenic mediators (15). This might be useful to properly restore bone dimensions in preparation for naturally appearing dental prostheses. The null hypothesis tested was that the functionalisation of PLGA membranes with the described method does not affect their bone regeneration efficacy.

## Material and Methods 

1.1. Preparation of the polymeric PLGA membranes

Sixteen 40-µm-thick, resorbable organic PLGA barriers based on poly(lactic-co-glycolic) acid copolymer were fabricated from a 1.5 wt% PLGA dichloromethane solution by evaporation of the solvent on a teflon plate at the Institute of Materials Science of Seville (ICMSE, Spain). The membranes were used to cover the bone defects prepared on the parietal bone of eight experimental rabbits’ skulls. Two groups of regenerative membranes (n = 2 each) were prepared and tested for GBR processes: 1: PLGA (control) and 2: PLGA/PO2/SiO2. The thickness of the SiO2 coatings ranged from 20 to 40 nm.

The control group was characterised by X-ray photoemission spectroscopy (XPS) so that 54% carbon and 46% oxygen were registered on the membranes’ surfaces. The molecular weight of the PLGA copolymer was 12 kDa. The PLGA was synthesised by means of the ring-opening copolymerisation of two different monomers, the cyclic dimers (1,4-dioxane-2,5-diones) of glycolic acid, and lactic acid. During polymerisation, successive monomeric units (of glycolic or lactic acid) were attached in PLGA by ester linkages, thus yielding linear, aliphatic polyester. The C1s spectrum of the bare PLGA consisted of three well-defined bands at 284.6, 287.5 and 288.5 eV; these bands are attributed to C-C/C-H, COO, and the COR functional groups of the composite polymer.

In the Group 2, the treatment of PLGA substrates with PO2 took place at close to ambient temperatures (RT, room temperature: 23.0 ± 1.0 ºC) and did not affect their structural integrity. The membranes’ surfaces were exposed to pure oxygen plasma in a low pressure radio frequency (RF) parallel plate reactor working at 13.56 MHz and 10 W for 1 min. This physical/etching technique improved the surface roughness of the barriers and favoured the adhesion of future coatings. The membranes were then covered with thin bioactive layers of SiO2 with nominal thickness of 20-40 nm (by means of ‘dry way’ procedures) to prevent contact between the polymer and liquid. This process was carried out at RT, by Plasma-Enhanced Chemical Vapor Deposition (PECVD) in a remote configuration system that consisted of an external microwave plasma source (SLAN, Plasma Consult, GMbh, Germany) separated 10 cm from the reactor chamber by a grounded grid to avoid the microwave heating of the barriers. The reactor was operated at 400 W under a total pressure of 4 × 10-3 Torr during deposition, with pure O2 as plasma gas, and hexamethyldisiloxane (HDMSO) as a precursor. Some of the co-authors patented this new formula for GBR processes (González-Padilla D, García-Perla-García A, Gutiérrez-Pérez JL, Torres-Lagares D, Castillo-Dalí G, Salido-Peracaula M, *et al.* (inventors): Membrana reabsorbible para regeneración ósea. Spanish patent: *P*-0201232018, 2012). A diagram of this set of procedures was shown in our pilot study (3).

1.2. Animal experimentation specimens

An a priori power calculation made from the data obtained in the pilot study (3) provided the sample size required to achieve statistical significance in the main investigation (α = 0.05, β = 0.2). Thus, eight white, male, New Zealand-breed experimentation rabbits with identical characteristics (age: 6 ± 0.5 months; weight: 3.5 ± 0.5 kg) were selected for the study and fed daily with rabbit-maintenance Harlan-Teckland Lab Animal Diets (2030). Four dental surgeons, a veterinarian, a supporting anaesthesiologist, a biologist, nurses, and specialised clinical assistants participated in the surgical interventions, which were carried out at the Minimally Invasive Surgery Centre Jesús Usón (CCMI, Cáceres, Spain) in September 2015 (time of day: 9 a.m.). This experiment complies with the World Medical Association (WMA) Statement on Animal Use in Biomedical Research (adopted by the 41st World Medical Assembly celebrated in Hong Kong in 1989, revised by the 57th WMA General Assembly celebrated in South Africa in 2006, and reaffirmed by the 203rd WMA Council Session celebrated in Buenos Aires in 2016); with the EU Directive 2010/63/EU about the protection of animals used for scientific purposes; with the EU Directive 86/609/EEC regarding the care and use of animals for experimentation; with the National Institutes of Health guide for the care and use of Laboratory animals (NIH Publications No. 8023, revised in 1978); and with the Spanish laws and regulations. This research obtained the approval of the Ethics Committee of the University of Seville (Spain). As stated by the legislative framework, the minimum number of animals was utilised for ethical reasons.

1.3. Surgical procedure

**Figure 1:**
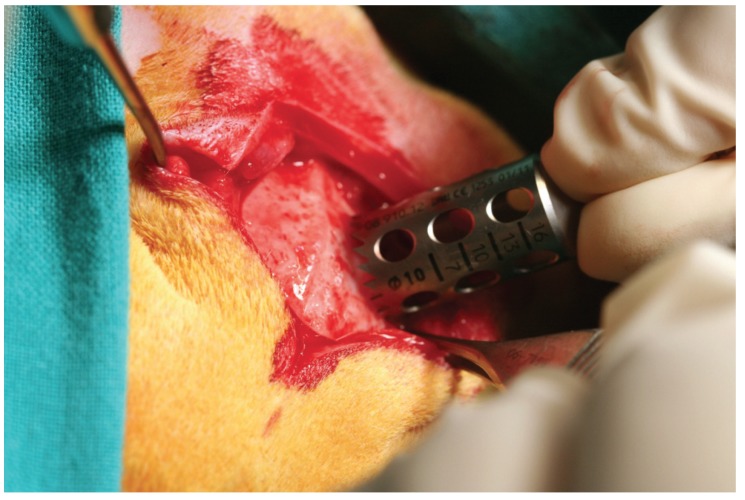
Intraoperatory picture.

The animals were first immobilised, and their vital signs were checked. In order to avoid animal pain and suffering, intravenous midazolam (0.25 mg/kg), and propofol (5 mg/kg) were administered as anaesthetics for induction. For maintenance, the animals inhaled 2.8% inspired sevoflurane gas. Analgesia was provided with ketorolac (1.5 mg/kg), and tramadol (3 mg/kg). Once the rabbits were sedated and prepared, both incisions between the bases of their ears, and between their eyes, were performed with a No. 15 scalpel blade and were connected with another incision made on the skull midline, so that a triangular field was discovered. The epithelial, connective, and muscular tissues were displaced using a Pritchard periosteotome. The skull surface was washed with a sterile saline solution with aspiration. Two bone defects (diameter: 11 mm; depth: 3 mm) were created on the parietal bone, on each side of the skull midline, 3 mm apart, using a trephine (Ref. 08.910.12, Helmut-Zepf Medical Gmbh, Seitingen-Oberflacht, Germany) mounted on an implant micromotor operating at 2,000 rpm under saline irrigation (Fig. 1). According to the manufacturer, the trephine characteristics were: internal diameter of 10 mm; sheath of 2.35 mm × 30 mm in length; and 19 teeth graduation. As the defects were created in a symmetric fashion in relation to the longitudinal axis of each skull, possible between-groups variations in section thickness were minimised. For each defect, the outer table and the medullar bone were completely removed with piezosurgery, and the inner table was preserved to avoid damage to the brain tissue (5). The depth was controlled with a periodontal probe, while the thickness of the bone pieces was measured with a millimetre rule.

**Figure 2:**
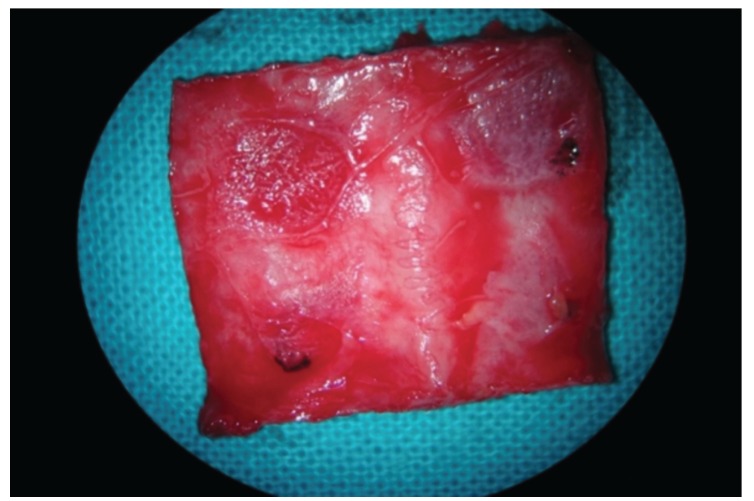
Regenerated calvarial bone in the experimental group. Bone sample extracted from the skull after the healing period.

One membrane of each group was randomly assigned to cover the bone defects of each rabbit. The randomisation sequence was generated using specific software (Research Randomizer, V. 4.0, Urbaniak GC & Plous S, 2013; http://www.randomizer.org/). The PLGA fibres of the barriers were also randomly oriented. The membranes were fixed with the fibrin tissue adhesive Tissucol (Baxter, Hyland S.A. Immuno, Rochester, MI, USA), which was placed on the bone rims adjacent to the defects. Proper adhesion and limited mobility of the membranes was confirmed when the flaps were moved back to their initial positions. Sutures were made on the following planes using resorbable material: periosteal (4/0), sub-epidermal (4/0), and skin (2/0). Simple stitches were used as close as possible to the edge. The operation lasted for about 1 h per specimen. The wound was carefully cleaned with a sterile saline solution. Anti-inflammatory analgesia (buprenorphine 0.05 mg/kg, and carprofen 1 ml/12.5 kg) was administered. The animals were sacrificed two months after surgery using an intravenous overdose of potassium chloride solution. Samples were obtained from the skull of each rabbit, cutting them in an anatomical sagittal plane (Fig. 2). After the brain mass was separated and the skull was washed with a sterile saline solution, the tissue samples were cut, marked individually, and set in 70% alcohol before being handled in the laboratory of histology. More illustrative images of the surgical procedure can be found in the latest publications of our group (3,14).

1.4. Processing of samples

The skull samples were processed for histological analysis at the Dental School of the Paris-Descartes University (1EA 2496, Montrouge, France). The protocols of Gallina *et al.* (12) and Torres-Lagares *et al.* (13) were strictly followed to complete the tissue fixation. After immersion in alcohol, the skull samples were fixed in a cold (4 ºC) 70% ethanol solution. Once dehydrated, the samples were embedded without demineralisation in methyl methacrylate (MMA) blocks (Merck, Darmstadt, Germany) which were set horizontally and perpendicularly to the sagittal axis, and polished in a conventional machine. Histological 5-µm-thick sections were cut using the Jung Ultrafrase Polycut E microtome (Reichert-Jung K, Leica, Heidelberg, Germany). To minimise the variance in thickness among the specimens, the slices were obtained from the middle region of the bone defects in series of ten slices each, placed on glass sample holders pre-treated with albumin, and set in 80% ethanol. The original bone disks were prepared for histology.

1.5. Staining and morphometric study

To evaluate the percentages of new bone formation, a metachromatic dye for histological staining and rapid contrast tissue analysis (Merck Toluidine Blue Merck, Darmstadt, Germany) (TB) was used. A 1% toluidine blue (TB) solution with a pH of 3.6 was adjusted with HCl 1 N. The samples were exposed to the dye for 10 min at RT, rinsed with distilled water, and air-dried. The von Kossa (VK) silver nitrate technique (Sigma-Aldrich Chemical Co., Poole, UK) was applied to visualise the mineralised bone.

To quantify the length of mineralised bone formed in the grown defects, intravenous labelling with calcein/demeclocycline in a 5% physiologic serum was carried out one week and one day before sacrifice. The reactives were calcein disolved into 2% NaHCO3 in serum (ref. SIGMA C0875 1 g, Sigma-Aldrich, USA), and demeclocycline hidroclorhide (ref. SIGMA A6140 5 g). The administration included 30 mg/kg of calcein (using 9 mg calcein/mL) for each rabbit (i.e., 45 mg for an animal of 1.5 kg) eight days before sacrifice. For each rabbit, 45 mg calcein was combined with 5 mL physiologic serum and 0.1 g NaHCO3; the VT was 5 mL per animal. The administration also included 30 mg/kg of demeclocycline (using 9 mg demeclocycline/mL) for each rabbit (i.e., 45 mg for an animal of 1.5 kg) one day before sacrifice. For each rabbit, 45 mg demeclocycline was combined with 5 mL physiologic serum; the VT was 5 mL per animal. Each rabbit was intraperitoneally injected with 5 mL of the solution. The calceine supplied a green colour to the new bone, and the demeclocycline provided a yellow colour.

To evaluate the resorptive activity of the regenerated bone tissue, the enzymological technique for evidence of tartrate of sodium resistant acid phosphatase (TRAP) was developed. This hydrolase served as a cytochemical marker for osteoclasts and pre-osteoclasts released into the surrounding environment during bone resorption to facilitate bone dissolution (16). The samples’ slices were incubated in Naphthol ASTR phosphate (Sigma-Aldrich), which was previously dissolved in NN dimethylformamide. Then, a 0.1 M buffer of acetate sodium tartrate and Fast Red TR salt hemi (zinc chloride) (Sigma-Aldrich) were added, the pH was adjusted to 5.2, and the final solution was filtered. The slices were incubated at 37 ºC for 1 h in the dark.

To investigate the ostheosynthetic activity of the regenerated tissues, the technique of evidence of alkaline phosphatase (ALP) was applied for osteoblasts and pre-osteoblasts. The slices were pre-incubated for 10 min in 0.1% Tris Triton (Sigma-Aldrich), washed in 0.1 M Tris hydrochloride, and incubated in the dark at 37 ºC for 30 min in Naphthol ASTR phosphate together with the coloured reaction developer Fast Blue RR Salt (Sigma-Aldrich) as a coupler (with adjusted pH = 9). A weak colouring of 1% TB was diluted to 10% in distilled water to give the samples contrast. Cells marked in violet, which defined the dimensions of the samples’ osteogenic strip, were considered as positive results.

As described in the previous subsection, the TRAP and ALP staining were performed on resin (methylmethacrylate) embedded samples to achieve better preservation of the trabecular bone structure (17-19). An improved MMA embedding method was applied (18), thus circumventing the decalcification procedure (19). These were the differences with respect to the original method: (a) Instead of dehydration of the bone samples with acetone (17), we used a standard dehydration protocol. Overall, acetone fixation causes massive shrinkage artefacts and acetone dehydration results in inferior quality of the bone section. (b) The infiltration protocol allowed achieving optimal infiltration of the tissue with the plastic. Good infiltration is the basis for uniform polymerisation and high section quality. (c) The addition of methylbenzoate to the methacrylate solution during infiltration and polymerisation improved preservation of antigenicity of the tissue. (d) Methylbenzoate acts as a plasticizer in MMA embedding mixtures. (e) The amount of the accelerator N,N-dimethyl-p-toluidine used for chemical polymerisation at low temperatures has been reduced, as recommended (18), resulting in a lower tendency for bubble formation and more homogeneous polymerisation of the blocks. (f) This procedure does not require purification and destabilization of the methacrylates used for infiltration and polymerisation (17-19).

The morphometric study of the bone was carried out by means of a Zeis Axioscop II microscope (Axioscop 40, Goettingen, Germany) using 4×, 10×, and 20× lenses. Pictures were taken with a digital signal processor (DSP) 3CCD camera (Sony, Tokyo, Japan), and Intelicam 8.0 software (Smart Matrox Imaging, Montreal, Canada). To quantify the newly regenerated tissue and to compare it with the original bone tissue, an assessment from distal (external) to proximal (internal) was made using ROI (region of interest) colour analysis with specific software (Fiji Is Just ImageJ, Tokyo, Japan). The following parameters were assessed by a different, blinded operator: percentage of new bone formation, length of mineralised bone formed daily in the grown defects (mm/day), concentration of osteoclasts (number of osteoclasts/mm2), and intensity of bone ostheosynthetic activity.

1.6. Statistical analysis

Mean and standard deviations (SD) were calculated for each quantitative parameter. The intra-examiner reliability was examined using the Kappa test (20). Since the Kolmogorov-Smirnov test demonstrated that the data were not normally distributed, the non-parametric Kruskal-Wallis test was run for post hoc comparisons (14). The level of significance was set in advance at a = 0.05 (12,13,16,20). The Statview F 4.5 Macintosh software (Abacus Concepts, Berkeley, CA, USA) was utilised for the analysis (3).

## Results

**Figure 3:**
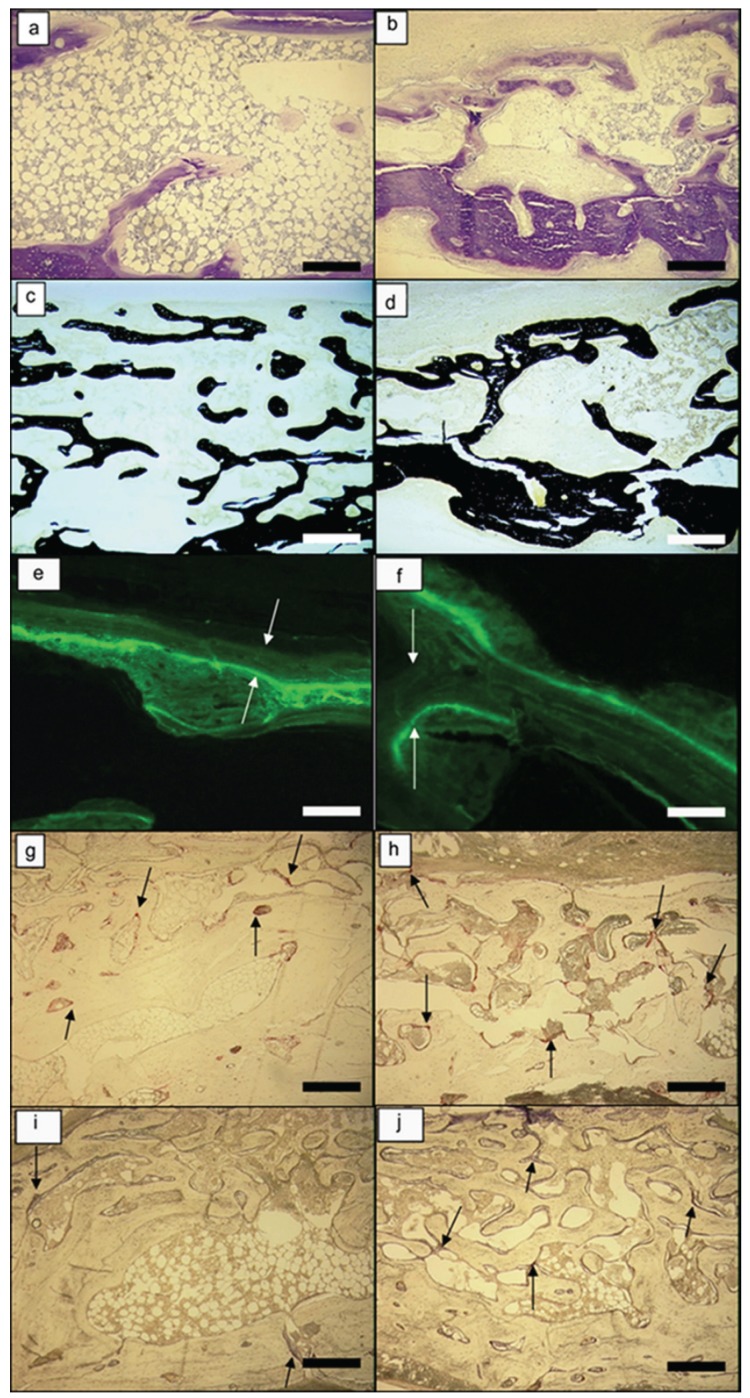
Morphometric analysis made under microscope at 2 months of follow-up. (a) TB image of the control group showing the lowest proportion of new bone formation. (b) TB image of the study group exhibiting bone in an advanced stage of formation. (c) VK image of the control group. (d) VK image of the study group confirming a larger formation of dense bone (Figs.: 2a-d; objective: 4×; bar: 250 µm; zoom: 1.5). (e) Calcein image of the control group. (f) Calcein image of the experimental group containing higher length of mineralised bone between the bone edge and the green line (Figs. 2e,f; objective: 20×; bar: 50 µm; zoom: 1.5). (g) TRAP image of the control group. (h) TRAP image of the study group with higher concentration of osteoclasts. (i) ALP image of the control group. (j) ALP image of the study group evidencing the highest ostheosynthetic activity (Figs.: 2g-j; objective: 5×; bar: 250 µm; zoom: 1.5).

For each experimental group of membranes, all of the animals were included in each analysis (8/8). The implanted biomaterials of both groups tested were well tolerated by the surrounding soft tissues, with no evidence of allergy symptoms, immune reactions, necrosis, or incompatibility after the healing period. The main study findings are illustrated in  Table 1, and in figure 3(a-j) . All of the images correspond to the superficial part of the regenerated bone. As the surgeon removed all of the bone except for the inner table (i.e., the outer table and the medullar bone were completely removed), the images show the new bone formed in a cranio-caudal direction. The Kappa statistic shows a perfect intra-examiner reliability (k = 1) for all of the evaluations performed (percentage of new bone formation, length of mineralised bone formed, concentration of osteoclasts, and intensity of ostheosynthetic activity).

**Table 1:**
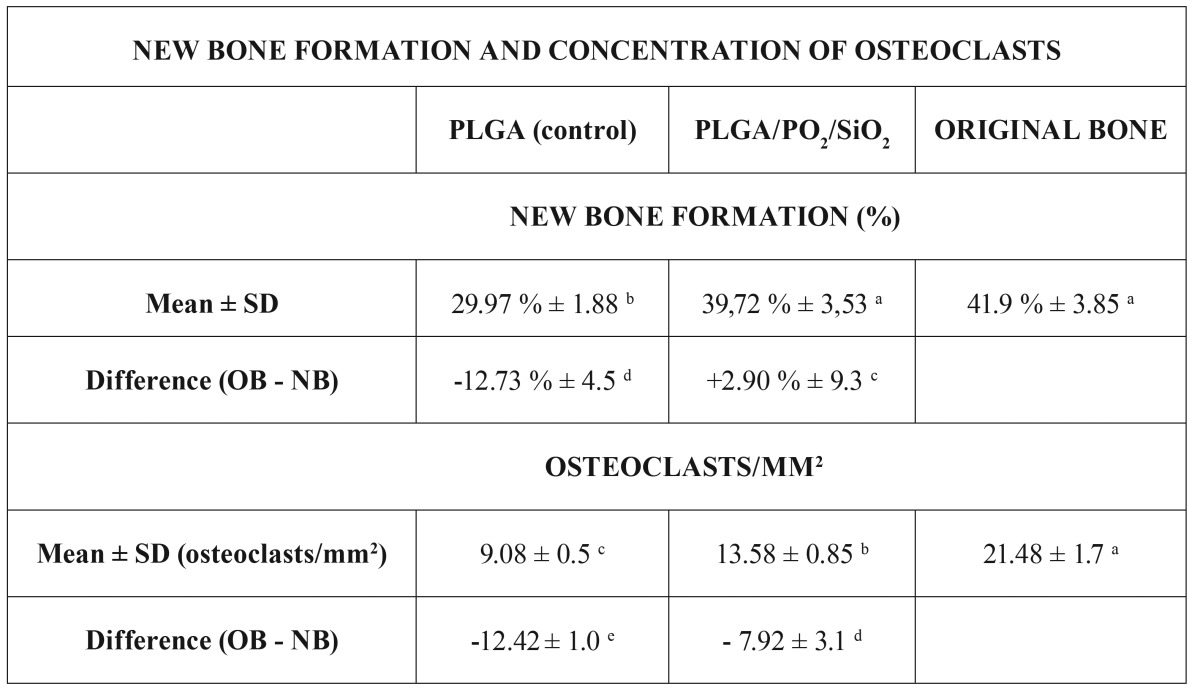
New bone formation and concentration of osteoclasts.

The PLGA/PO2/SiO2 barriers confirmed higher proportion of dense and thick endocranial and exocranial cortical bone tissue when compared with the control group in the histological slices (Fig. 3a,b). Moreover, the study group and the original bone showed similar proportion of dense cortical bone tissue. Marrow and adipose-like tissues were more detectable in the untreated PLGA scaffolds, whereas the PLGA/PO2/SiO2 membranes exhibited only a little adipose-like surrounding tissue. The histological evaluation therefore suggested some association between the appearance of this kind of tissue and the modification of the membranes’ surfaces.

In the TB analysis, the control group demonstrated less formation of new bone than did the PLGA/PO2/SiO2 group (Fig. 3a,b). As a result of the VK test, the experimental group achieved significantly higher percentage of new bone formation than did the controls (*p* = 0.039) (Fig. 3c,d). In this aspect, the functionalised group and the original bone were comparable, meaning that the control group showed the significantly lowest formation of new bone in the study (*p* < 0.05) ( Table 1).

The study group evidenced higher length of mineralised bone formed in the grown defects when compared with the control group, as it was interpreted from the calcein images (0.065 ± 0.015 mm/day vs. 0.032 ± 0.005 mm/day, respectively; *p* = 0.034) (Figs. 3e,f). In these images, the distance between lines was measured. The green line (injected eight days before euthanasia) is very clear, while the yellow one (injected 24 h before euthanasia) is faint. Hence, the bone edge was used instead to make the calculations.

With regard to the TRAP analysis, the experimental group exhibited significantly higher concentration of osteoclasts than did the control group (*p* = 0.0039) (Fig. 3g,h). Nonetheless, the original bone tissue demonstrated significantly higher concentration of these cells than did the PLGA/PO2/SiO2 group (*p* = 0.005) ( Table 1). Unlike the control group, both the study group, and the original bone tissue showed more than 2 osteoclasts per mm2.

The ALP technique attributed the highest ostheosynthetic activity to the study group. The blue linear distribution of osteogenic cells on the bone surface was more evident in the modified membranes (Fig. 3i,j).

## Discussion

Our results require the rejection of the null hypothesis, as the functionalised barriers demonstrated significantly higher bone regeneration potential than did the simple PLGA membranes when assessed as the combination of formation of new bone, length of bone formed daily in the grown defects, concentration of osteoclasts, and intensity of ostheosynthetic activity ( Table 1, Fig. 3).

The PLGA-based scaffolds were easy to use and handle, and showed no displacement after implantation. These membranes demonstrated high biocompatibility, since no immune reaction, symptomatology of allergies, abnormal swelling, intolerance by the underlying soft tissues, or evidence of necrosis was detected in any group (3,14). There is enough evidence in the literature remarking that the results of bone regeneration are worse in the absence of a GBR membrane (5,21,22). Incomplete bone formation (21), lower height of the restored bone, and bone defects partially filled by the overlying soft and dense fibrous tissues (5,22) are frequently found in these cases. Simple PLGA barriers were thus selected instead as the control group. Furthermore, the treatment of PLGA membranes with oxygen plasma (PO2) clearly improved the histological results when compared with those barriers that were directly coated with inorganic films of silicon dioxide particles (PLGA/SiO2) in our pilot study (3). While direct deposits of SiO2 particles seem to generate a flattening effect (3,23), the physical pre-treatment with PO2 increases the surface roughness of the PLGA biomaterial (23); which promotes the adhesion of osteogenic mediators. This is crucial, since osteoblasts and other molecules that grow anchored to an extracellular matrix (ECM) require to be bonded to the substrate before starting a normal function (24). The roughening effect generated by the plasma treatment allows signalling from the ECM to nucleus via integrins, cytoskeleton, and molecular cascades (i.e., G-proteins, kinases, and ions). This attraction of osteogenic cells and particles also assists the degradation of the barriers, thus favouring the bone regeneration (8,23). The effect of the plasma treatment itself (which was not the focus of our experiment) has been extensively documented (8,23). Even though the PO2 was basically applied in the study group to prepare the membranes’ surfaces for improving the retention of the SiO2 particles, it might have somehow affected the bone regeneration process (3,14).

The stimulating effect of silicon on the proliferation, and differentiation of osteoblastic cells has been confirmed (9,10,15). Silicon has been described as an important element of osteogenesis, as it seems to enhance the osteoblasts’ potential for reparation and mineralization in the damaged tissues where it is implanted (25). Recent studies have even demonstrated that through providing osteoporotic patients with silicate supplements instead of biphosphonates, a major increase of the femur and back bone density levels may be observed (25,26). SiO2nanocomposite particles could therefore act as primer initiators for the synthesis of a mineral bone matrix (15), expediting the healing and repair of bone defects (14). Even though the effect of this new material on the differentiation of osteoblasts by the regulation of osteo-specific genes involved in the neogenesis of the mineralized matrix deserves further investigation (24), in this research, the combination of oxygen plasma and silica oxide apposition for the functionalisation of PLGA membranes supplied the best results of bone regeneration (Table 1; Fig. 3).

Concerning the histological analysis, dense layers of exocranial and endocranial mature bone in rather advanced stages of formation were found in those defects that were covered with PLGA/PO2/SiO2 membranes. Conversely, greater proportions of marrow and adipose-like tissues surrounded the bare PLGA barriers. This micromorphology is characteristic in immature tissues, retard phases, or initial-growing phases when the GBR membranes are not active enough (3). However, when the scaffolds are functionalised with active nanoparticles, such as SiO2, only a few marrow and adipose like tissues are detected (3,14) ( Table 1; Fig. 3a,b). Future studies should confirm this correlation between the type of barrier and the presence of immature tissues (7,27,28).

Overall, the advantages of PLGA membranes mainly depend on their structural design and topography; which may increase their adhesive potential, enhance the osteoblasts’ ability to migrate and bond at the cell-substrate interphase, and help direct and organise the adhesion and differentiation of bone cells to facilitate proper tissue repair (29). The calcein test yielded the most conclusive evidence in this regard, revealing that the lineal rate of newly mineralised bone formed daily in the grown defects (30) doubled when PLGA/PO2/SiO2 membranes were used (Fig. 3e,f). The TB (Fig. 3a,b), and the VK analysis (Fig. 3c,d) attributed the highest percentage of new bone formation to the PLGA/PO2/SiO2 scaffolds, which showed comparable levels to those of the original bone tissue ( Table 1). Two months after surgery, the process of bone healing was still active, and the remodelling phase became noticeable. The TRAP test (Fig. 3g,h) confirmed the existence of multinucleated giant cells (which act as energy reserves for trauma regeneration) (28), mainly in the case of the functionalised barriers. The TRAP analysis detected a significantly higher concentration of osteoclasts in the functionalised group when compared to the controls. However, the original bone exhibited the highest number of osteoclasts/mm2 in the study ( Table 1). Finally, the ALP analysis confirmed higher ostheosynthetic activity when the treated membranes were used to cover the bone defects ( Table 1; Fig. 3i,j). This fact, which is inherent in regenerative processes (3), was an indicator of the osteoconductive capacity of the SiO2 nanocomposite particles, and revealed the migration of osteoblasts to the inner defects (28). For ensuring an adequate osteosynthesis, bone must be continuously destroyed and regenerated so that a proper equilibrium of the activity of osteoblasts and osteoclasts is achieved (14). Thus, PLGA/PO2/SiO2 membranes confirmed greater bone regeneration capacity than did PLGA barriers in view of the results of the four variables tested ( Table 1; Fig. 3).

These four variables disclosed comparable results in a related animal experiment that compared functionalised PLGA/PO2/TiO2 GBR membranes with the same controls (14). Notwithstanding that either SiO2 or TiO2 nanocomposite particles could act as exciters of the surrounding osteoblast and pre-osteoblast cells; the induction of changes in nuclear morphology, gene transcription, and cells’ phenotypic expression caused by each type of inorganic coating, and the way in which this fact may contribute to bone regeneration in the long term still remain uncertain and should be examined and contrasted in future investigations.

Apart from the limitations that are inherent to any animal model when the tested material is designed for being utilised in humans, the use of parietal bone may be the main limitation of this study. The sample size was previously calculated in order to reduce the number of animals for ethical reasons. Moreover, the membranes were randomly assigned to the bone defects, and a blinded operator performed the morphologic study. Despite the fact that the apposition of SiO2 nanoparticles onto PO2 pre-treated PLGA membranes seems to be a promising new formula for GBR processes that could help in guaranteeing appropriate support and aesthetics of our prosthodontic rehabilitations, these barriers should be tested in human maxillary bone before being recommended.

The following conclusions may be drawn from the present study: (1) the incorporation of silica dioxide layers onto PLGA membranes modified with PO2 improves the results of bone regeneration in both quantity and quality when applied to skull defects in an animal model; (2) the addition of SiO2 nanoparticles to PLGA membranes’ surfaces stimulates the new bone formation, mineralisation, and ostheosynthetic activity, when compared to simple PLGA scaffolds (actually, the functionalised substrates promoted similar degrees of new bone formation than those of the original bone tissue); (3) together with the requisite of more animal studies, further steps should be taken for investigating the application of these modified membranes in humans to restore bone dimensions, thus optimising the functional features and the natural appearance of our future prosthetic restorations.
